# Evaluation of electrode-sample contact impedance under different curing humidity conditions during measurement of AC impedance of cement-based materials

**DOI:** 10.1038/s41598-020-74925-w

**Published:** 2020-10-21

**Authors:** Ruipan Wang, Fuqiang He, Changping Chen, Lizong Dai

**Affiliations:** 1 College of Materials, Xiamen University, Xiamen, Fujian 361005 People’s Republic of China; 2 School of Civil Engineering and Architecture, Xiamen University of Technology, Xiamen, Fujian 361024 People’s Republic of China

**Keywords:** Civil engineering, Techniques and instrumentation, Characterization and analytical techniques, Engineering, Materials science

## Abstract

In this study, a simple method was proposed to calculate electrode-sample contact impedance in the cases of two-point and four-point measurements. The results indicated that when using the saturated calcium hydroxide solution (SCH) as conductive medium, the contact impedance in the four-point measurement is negligible for the impedance range of cement-based materials. The SCH can be used as a reference for correction of the contact impedance. A reasonable combination of curing humidity and different conductive media is recommended for the two-point measurement, which is suitable for testing the ACIS of cement-based materials. In a case of contact impedance not being precisely known, it is highly recommended that a four-point measurement with two different ratios of the length of the sample and the center spacing of the voltage electrodes (*L*/*a*) should be conducted to evaluate the effect of the contact impedance following the procedure proposed in this study.

## Introduction

Since AC impedance spectroscopy (ACIS) was first used to analyze cement-based materials^1^, the ACIS has been widely used for testing their microstructure and properties^2–4^. ACIS can be obtained using two-, three-, and four-point measurements. Up to now, many researchers have used two-point measurements to get the ACIS of the cement-based materials. However, whether the contact impedance in two-point measurements has a large effect on the ACIS is controversial. Ford et al.^5^ found that the material impedance from the two-point measurement is more susceptible to the electrode-sample contact impedance than those measured by the three- and four-point measurements. Some researchers believe that when using a four-point measurement system, the polarization resistance of electrodes can be effectively eliminated^6,7^. However, Xie et al.^8^ found that the four-point measurement with point contact cannot reflect the true information about the hydrating cement system and that the two-point measurement can give more reliable results. At present, the contact impedance of the four-point measurement is widely accepted as negligible^9^. Hwang et al.^10^ suggested that the DC resistance of the four-point measurement can be used to evaluate the test results of the two-point measurement. However, there may be data fluctuations when conducting a DC test of cement-based materials^11^. McCarter et al.^7^ considered that the true impedance of the sample can be obtained by a four-point measurement and found that the apparent resistance measured by two- and four-point measurements were very similar when a solution-soaked sponge was used as the conductive medium. Therefore, they believed that the contact impedance in two-point measurement can be ignored when using a highly conductive sponge-saturating liquid as a conductive medium between the electrodes and the sample. However, there may be a special case that cannot be ignored, that is, the contact impedance of the two-point measurement and the four-point measurement is at a similar and large value. However, the four-point measurement can minimize the contributions of impedance caused by contact, but it cannot be completely eliminated in theory as stated in the review by Miccoli et al.^9^, the results of the four-point measurements are also influenced by the homogeneity of the sample or the isotropy of each phase and these sample-specific characteristics and technical constraints such as the probe geometry and the size of sample, for the cement paste, they can be regarded as homogeneous materials. Additionally, the results of the four-point measurement essentially depends on geometry and are sensitive to the relative position of electrode in sample and boundary conditions^12–14^. Therefore, evaluation of the contact impedance in the four-point measurement becomes critical.

Many direct contact methods have been applied to the two-point measurement, such as precast electrode and end-contact methods^15,16^, and conductive media contact methods, such as cement slurry^17^, filter paper saturated with NaOH solution^18^, sponge immersed in NaOH solution, pore solution or deionized water^11^, conductive adhesive^19^, insulating materials^15,20^ and saturated Ca(OH)_2_ solution^21^, however, the effect of curing humidity conditions on the contact impedance has never been noticed. The contact impedance in a direct contact method is easily influenced by the curing humidity conditions of the samples^22^. Therefore, it is necessary to pay attention to whether the contact impedance in the conductive media contact method will be affected by the curing humidity conditions. In addition, although the precast electrode method can be used to accurately measure the impedance of the samples cured in case of humidity ≥ 95%, the contact impedance becomes unacceptable when the humidity decreases^22^. The precast electrode method is also not suitable for investigating some special situations such as ion migration in cement-based materials^23^, carbonization^2,24,25^, freeze–thaw of cement-based materials^26^, etc. When the precast electrode method is used in engineering, where the curing humidity condition is uncontrollable, it is very important to ensure that the contact impedance caused by the humidity condition is at a negligible level.

This paper therefore focuses on the evaluation of electrode-sample contact impedance under different curing humidity conditions during measurement of AC impedance of cement-based materials. Based on this purpose, this paper will mainly solve the following problems: (1) Although the electrode polarization can be eliminated in the four-point measurement^7,27^, the contact impedance composed of the interface impedance between the electrodes and sample, as well as the extended impedance due to the difference in cross-sectional area between the electrodes and sample still exists, but what is the real value? (2) What kind of conductive medium can be used to minimize the contact impedance based on consideration of the different curing humidity conditions? Finally, some considerations for reducing the contact impedance during two-point measurement are also discussed.

## Raw materials and experimental methods

### Raw materials

Ordinary Portland cement (P.O.42.5) according to Chinese Standard GB175-2007^28^ was used in this experiment, and its chemical composition is shown in Table 1.

**Table 1:** Chemical components of the cement (w%, by mass).

Cement	CaO	SiO_2_	SO_3_	Al_2_O_3_	Fe_2_O_3_	MgO	Na_2_O_eq_	f-CaO	Loss
*w*%	62.13	20.76	2.80	4.58	3.27	3.13	0.057	0.76	1.86

### Preparing and curing samples

**Figure 1:**
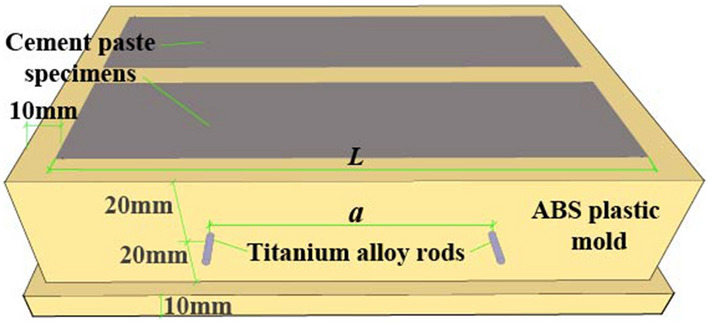
Schematic of sample with precast titanium alloy rod electrodes.

**Figure 2:**
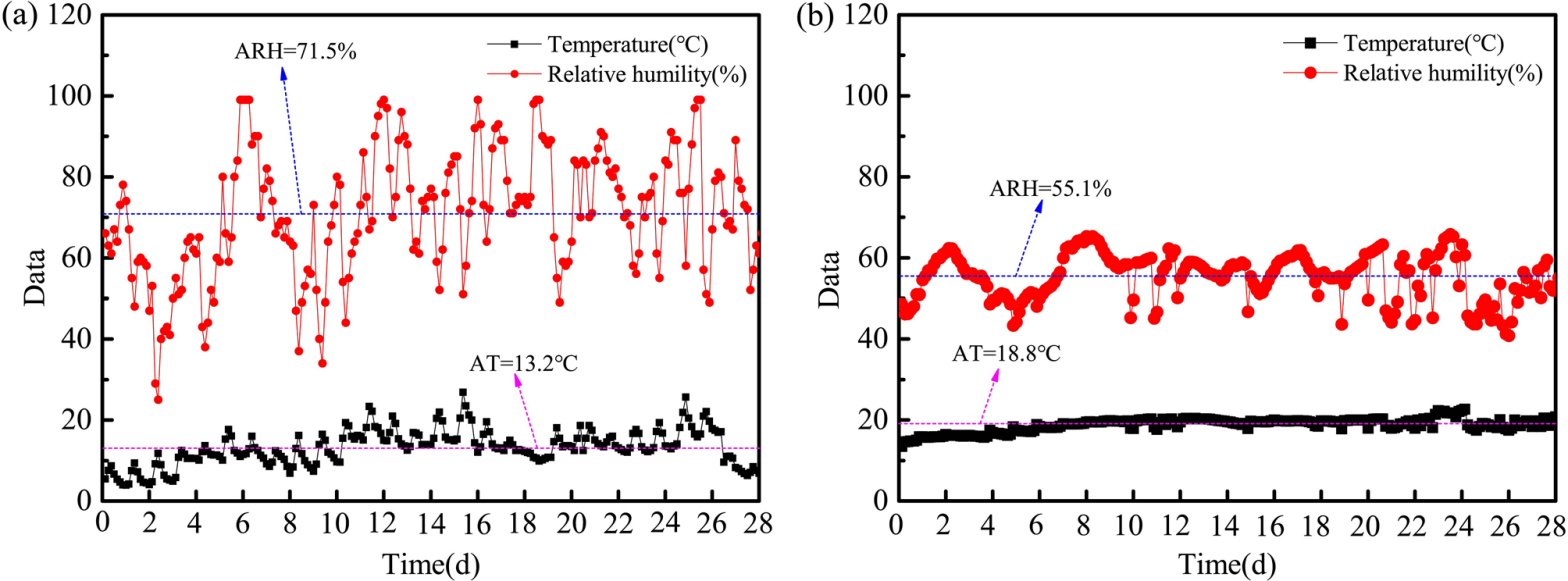
Temperature and relative humidity of the outdoor and indoor cuing environment. (**a**) Outdoor curing condition. (**b**) Indoor curing condition (AT: average temperature, ARH: average relative humidity).

The size of the sample used for the ACIS test was 40 mm × 40 mm × 160 mm, and the water-cement ratio of the sample was 0.35. The casting mold was made of ABS insulating engineering plastic. The electrodes arrangement in the four-point measurement was according to publication^7^. A schematic diagram of the sample with the precast titanium alloy rods as voltage electrodes is shown in Fig. 1, the samples in the mold are used for four-point measurement. The electrodes arrangement in the two-point measurement was described in detail in reference^22^. Samples were demolded after 1 d and placed under different curing humidity conditions, as shown in Table 2. Three samples were cast for each curing humidity condition, and the average of the three test results was taken as the final result for each condition. The temperature and humidity of outdoor curing and indoor curing conditions are shown in Fig. 2.

**Table 2:** Different curing conditions used in this study.

Curing methods	Description
Fog curing	Relative humidity (RH) ≥ 95%, Temperature (T) = (20 ± 2) °C
60% RH curing	RH = (60 ± 5)%, T = (20 ± 2) °C
Outdoor curing	Temperature and humidity varied as shown in Fig. 2a
Indoor curing	Temperature and humidity varied as shown in Fig. 2b
Water curing	Immersing samples in a water tank filled with tap water and the water tank was placed in a fog room
SCH curing	Immersing samples in a water tank filled with saturated Ca(OH)_2_ solution and the water tank was placed in a fog room
Oven-drying	Indoor curing for 28 d and then oven-drying at 60 °C for 48 h

### Experimental methods

ACIS were obtained for each sample using a Solartron 1260 impedance analyzer. The test frequency range was 10 MHz–1 Hz, as measured using a logarithmic sweep with 15 frequency points per decade, and the excitation voltage was 100 mV. The electrodes at both ends of the sample were 40 × 40 × 1.0 mm stainless steel electrodes. For the same sample, the order of use of the conductive medium was conductive glue (CG), the saturated Ca(OH)_2_ solution sponge (SS) and the saturated Ca(OH)_2_ solution (SCH). The main component of the conductive glue is inorganic aluminosilicate material, the sponge is a synthetic sponge with a size of 40 × 40 × 2.0 mm, and the Ca(OH)_2_ is analytically pure. The connection methods between the sample and electrodes at both ends of the sample through conductive medium in four- and two-point measurements can be found in^7,22^, respectively.

**Figure 3:**
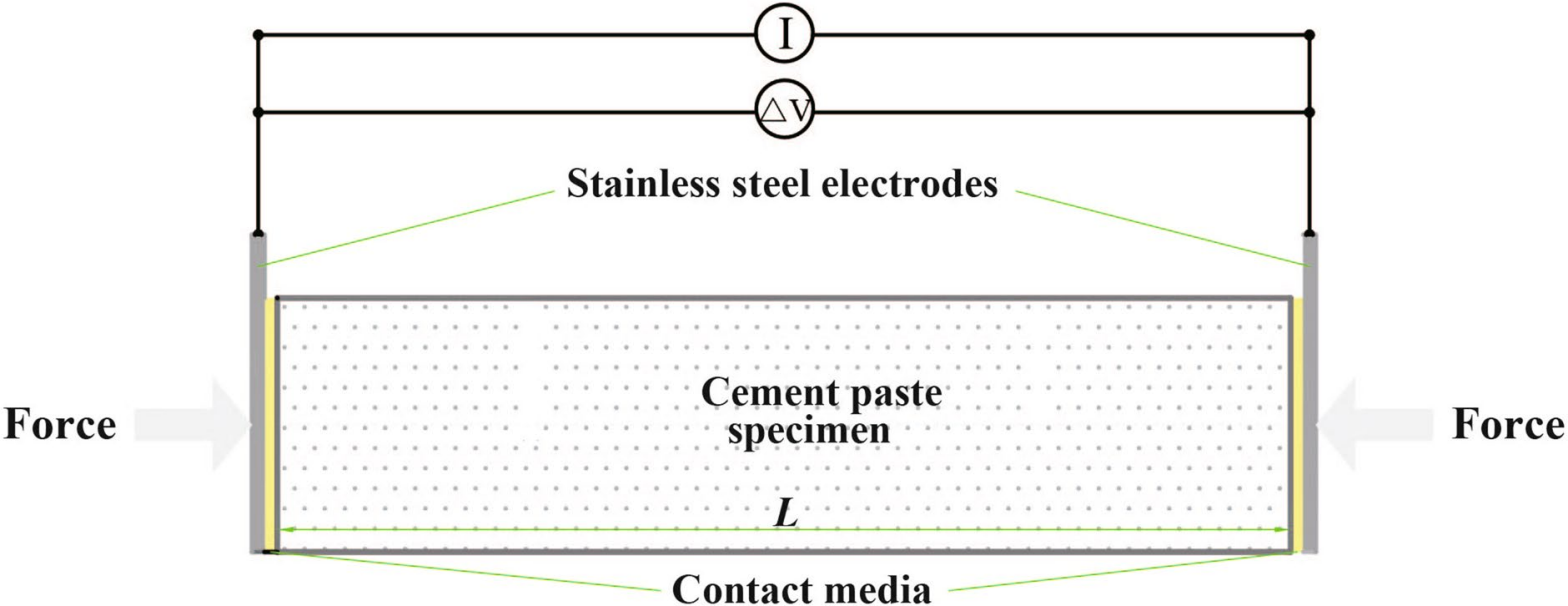
Cross-section of the two-point measurement.

**Figure 4:**
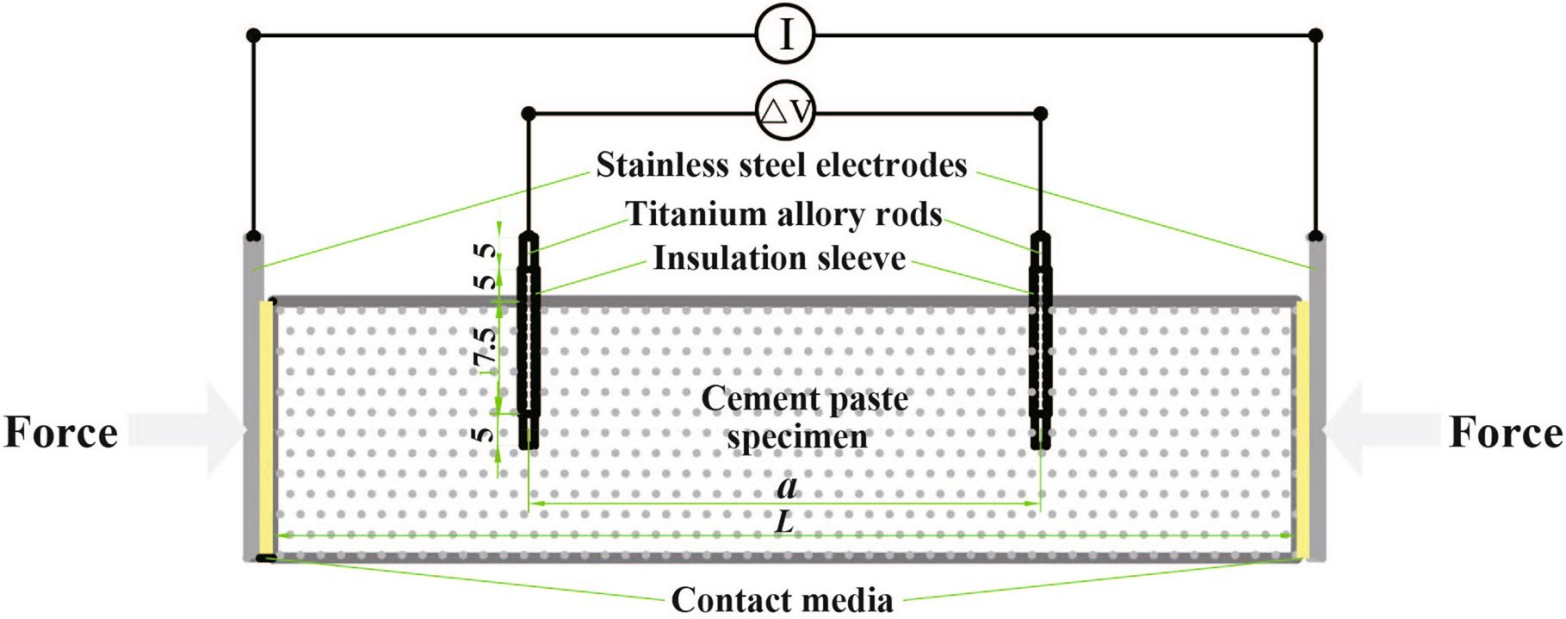
Cross-section of the four-point measurement (*L* represents the length of the sample, *a* represents the center spacing of the voltage electrodes).

Details of the two-point measurement using the SS as the conductive medium can be found in^7^. A pressure of 1.6 kPa on both sides of the electrodes was applied during measurement to ensure good contact between the electrodes and the sample^7,29^. Schematics of the two- and four-point measurements are shown in Figs. 3 and 4, respectively.

**Figure 5:**
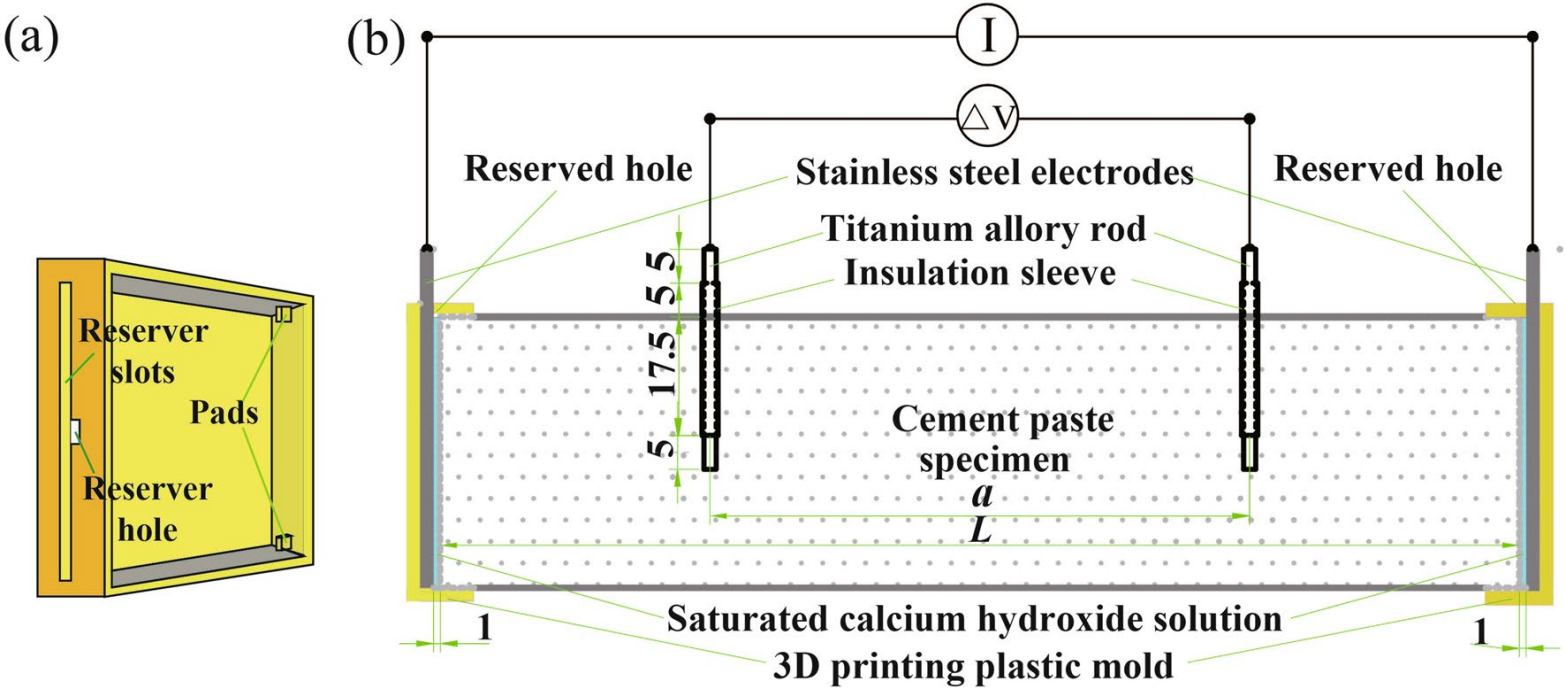
Schematic of the mold with SCH used as the conductive media. (**a**) 3D printing plastic mold; (**b**) Sectional view of sample for the four-point measurement.

When SCH was used as the conductive medium, the electrode arrangement was shown in Fig. 5. The 3D printed mold was used to store the solution, as shown in Fig. 5a. During the measurement, the connection between the printed mold and sample was sealed with glass glue to prevent the solution from leaking out. The distance between the end of the sample and the electrodes was 1.0 mm and the relative position was kept unchanged and maintained the solution leak free during the measurement.

## Fundamental theory for four-point and two-point measurement of AC impedance spectroscopy

### Conductive theory of two-point and four-point measurement of ACIS

In the two-point measurement, the excitation electrodes simultaneously act as working electrodes, the dissolved free ions in the cement paste or conductive media tend to move towards the electrode/sample interface under the influence of an electric field, leading to the formation of ionic double layers in the electrode-interface region. The interface reaction speed is slow, and the electric charge brought to the interface by electronic conduction cannot be transferred to the ionic conductor in time, so that the charge accumulates on the electrode surface^30,31^, indicating the generation of a huge polarization resistance of the electrode at low frequencies^32–36^, which is associated with a faradaic process and connected in series with the impedance of the sample^37^. However, as the frequency increases, the polarization resistance decreases until it vanish^38^, and is often considered negligible at sufficiently high frequencies. This is because when the frequencies are above certain values, as some researchers have suggested, in the interval 100–500 kHz^32^ or above 10 kHz (10^4^ Hz)^39^, the current at the interface flows via the double layer capacitance, without significant faradaic processes^6^. However, it is difficult to estimate an upper bound for this frequency limit^32^. In addition, although the contact impedance may only affect the bulk impedance within a certain frequency range, experimental results show that it does change the radius of the material's impedance response arc. In some cases, the change is quite significant (see “Selection of conductive media in the two-point measurement” section), which indicates that it is very necessary to evaluate the influence of the contact impedance on specimen impedance in different situations.

The four-point measurement techniques were first described by Schwan^40^ to eliminate the problem of electrode polarization by providing a second pair of electrodes, non-current-carrying, with which to measure the voltage across the sample^6,27,32,37,39,41,42^. However, a potential difference will occur between the internal voltage electrodes as current flows through the sample, there will be a current flowing through the voltage electrodes in theory, which is consistent with the principle of voltage measurement. Based on this theory, the internal voltage electrodes will also produce polarization resistance. In this case, the voltage electrode will generate interface impedance and extended impedance, which constitute the main part of contact impedance.

Many researchers^43–46^ simulated the electric field distribution in cement sample. B. Díaz et al.^43^ simulated the influence of cell geometry on the measurement of resistivity of the cement-based materials, and found that the effect of the electric field distribution is a function of the resistivity. When the areas of the sample and electrode are equal, the apparent resistivity is independent of the thickness of the sample. Therefore, the electrode of area same with the cross-sectional area of the sample was used in this study. In fact, the distribution of current in the sample during two-point and four-point measurements are very interesting, but it is not within the scope of this article, and will be studied in future research.

**Figure 6:**
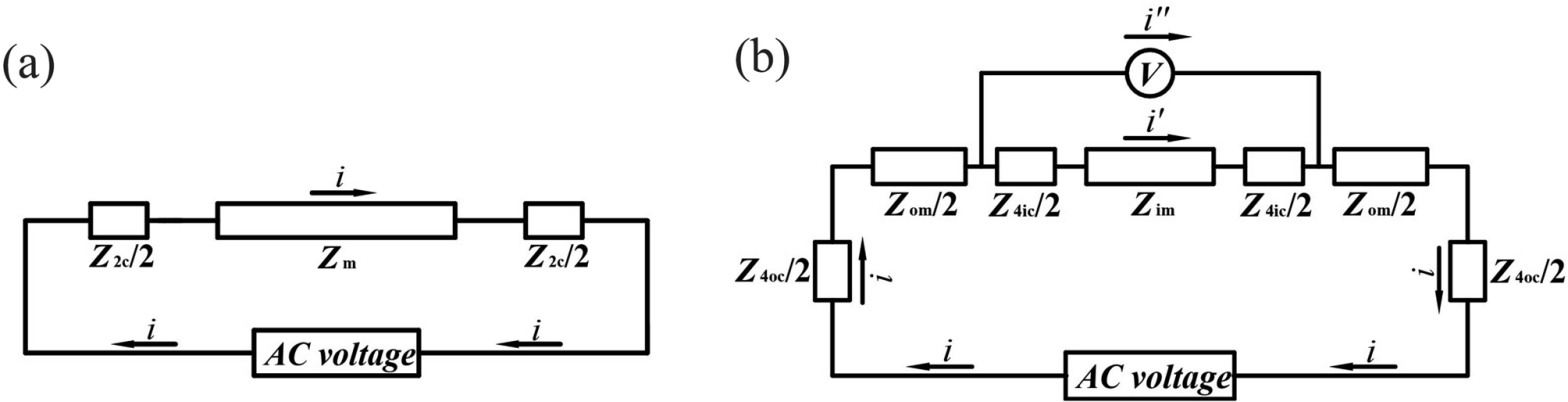
Circuit plots of AC impedance measurement. (**a**) Two-point measurement; (**b**) Four-point measurement.

Circuits for the two-point and four-point measurements are shown in Fig. 6. In this paper, we defined the contact impedance as the impedance caused by the connection between the electrodes and the sample, including the polarization resistance of the electrodes, the interface impedance between the electrodes and the sample, and the extended impedance caused by the difference in size between the electrodes and sample^10^. It can be seen from Fig. 6 that both two-point and four-point measurements contain contact impedance. Further, it can be seen from Fig. 6b that in the actual measurement, the contact between the voltage electrodes of the four-point measurement and the sample will also produce bias current *i*'', which can be neglected when the excitation current is very small^39^. However, the variation range of the contact impedance composed of the interface impedance^47^ and the extended impedance^10^ has not yet been clearly determined in the four-point measurement of cement-based materials. In fact, in some cases such as indoor curing and using CG as conductive medium, the four-point measurement does have a non-negligible contact impedance, as shown in “Evaluation of the contact impedance in the four-point measurement” section.

Where: *Z*_2c_ is the contact impedance between the electrodes and the sample in the two-point measurement; *Z*_m_ is the impedance of the sample; *Z*_4oc_ is the contact resistance between the current electrodes and the sample in the four-point measurement; *Z*_4ic_ is the contact impedance between the voltage electrodes and the sample in the four-point measurement; *Z*_om_ is the impedance of the sample outside the voltage electrodes in the four-point measurement; *Z*_im_ is the impedance of the sample between the two voltage electrodes in the four-point measurement; *Z*_t2_ is the total impedance in the two-point measurement; *Z*_t4_ is the total impedance in the four-point measurement; *i* is the total current flowing through the circuit during the four-point measurement; *i*’ is the current flowing through the sample between the voltage electrodes in the four-point measurement; *i*” is the bias current flowing through the voltmeter in the four-point measurement.

### Theoretical derivation of the relationship among the four-point and two-point measured impedance and the contact impedance

Based on the position of the electrodes in Fig. 4 and the circuit plots of the two- and four-point measurements shown in Fig. 6, Eq. (1) can be obtained:

1 $$Z_{m} = Z_{im} + Z_{om} { = }\frac{L}{a}Z_{im}$$

For the two-point measurement, the measured *Z*_t2,_ can be expressed as Eq. (2):

2 $$Z_{t2} = Z_{m} { + }Z_{2c}$$

For the four-point measurement, the measured *Z*_t4_ can be expressed as Eq. (3):

3 $$Z_{t4} { = }Z_{im} + Z_{4ic}$$

Multiplying Eq. (2) by *a* and subtracting Eq. (3) by *L*, Eq. (4) can be obtained:

4 $$a{\text{Z}}_{t2} - LZ_{t4} { = }a(Z_{m} + Z_{2c} ) - L\left( {Z_{im} + Z_{4ic} } \right)$$

Substituting Eq. (1) into Eq. (4), Eq. (5) can be obtained:

5 $$a{\text{Z}}_{t2} - LZ_{t4} { = }aZ_{2c} - LZ_{4ic}$$

**Figure 7:**
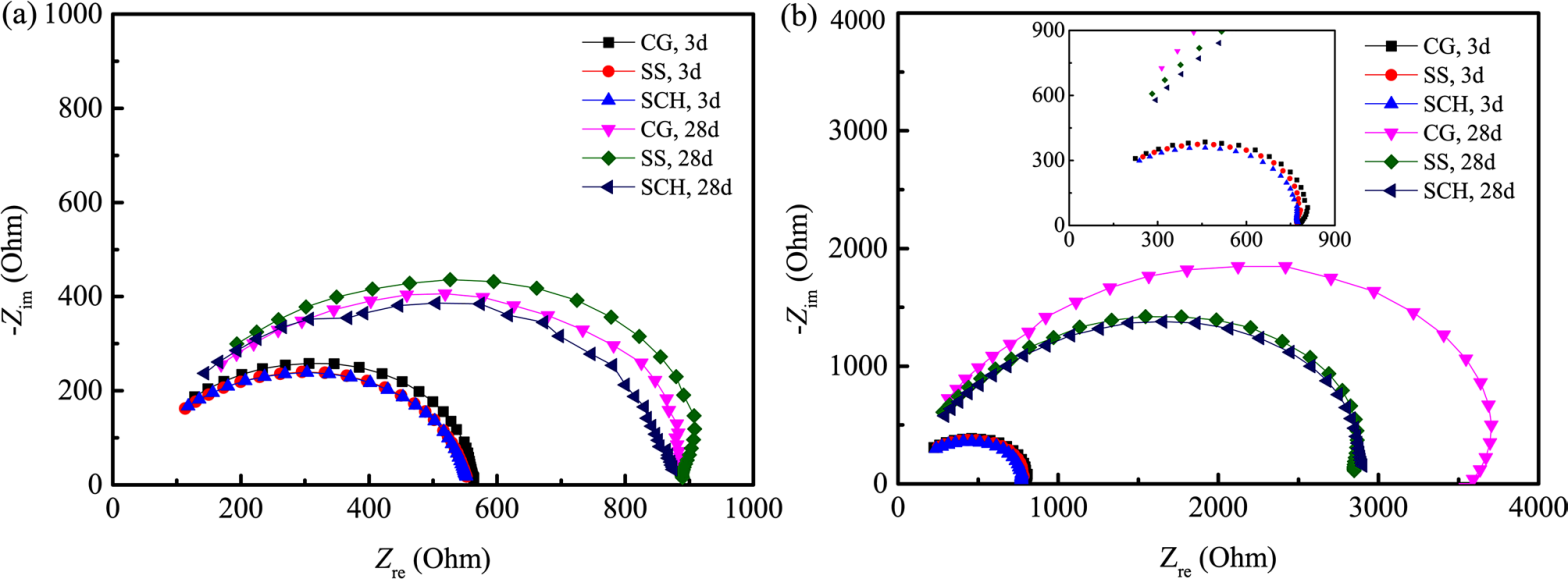
Nyquist plots of the four-point measurement with different conductive media between current electrodes and the sample (10^5^–10^7^ Hz). (**a**) In the fog room curing condition. (**b**) Indoor curing condition.

Multiplying both sides of Eq. (5) by 1/*a*, Eq. (6) can be obtained:

6 $${\text{Z}}_{t2} - \frac{L}{a}Z_{t4} { = }Z_{2c} - \frac{L}{a}Z_{4ic}$$

where the meaning of all symbols are the same as those in Fig. 7.

It can be seen from Eq. (6) that if the four-point measurement results are used to prove whether the contact impedance in the two-point measurement is negligible, it must be satisfactorily shown that the contact impedance in the four-point measurement is small enough to be negligible and that the *L*/*a* value is small enough. In addition, if the polarization resistance of the four-point measurement can be neglected, whether the interface impedance and extended impedance can also be neglected is a prerequisite of the universality of the four-point measurement, which will be discussed in “Discussion of the four-point measurement” section.

## Results and discussion

### Discussion of the four-point measurement

#### Comparison of measured results by different conductive media in the four-point measurement

Four-point measurements with *L*/*a* = 2 were conducted for samples cured in a fog room and indoor environment, with CG, SS and SCH used as the conductive media between the current electrodes and specimens, as the voltage electrodes were precast in the cement paste specimens. All ACIS are given in Fig. 7. It can be seen from Fig. 7 that the ACIS measured by the four-point measurement with different conductive media are very similar in high frequency regions for 3 days cured samples in a fog room and in an indoor curing condition. While the ACIS of samples cured for 28 days are different, especially for those of samples cured in an indoor environment, the high frequency arc in ACIS measured by CG is much larger than that seen for other conductive media. This indicates that conductive media have a completely different effect on ACIS in the high-frequency region of the four-point measurement and the CG has caused severe distortion of the ACIS of samples cured in an indoor environment.

**Figure 8:**
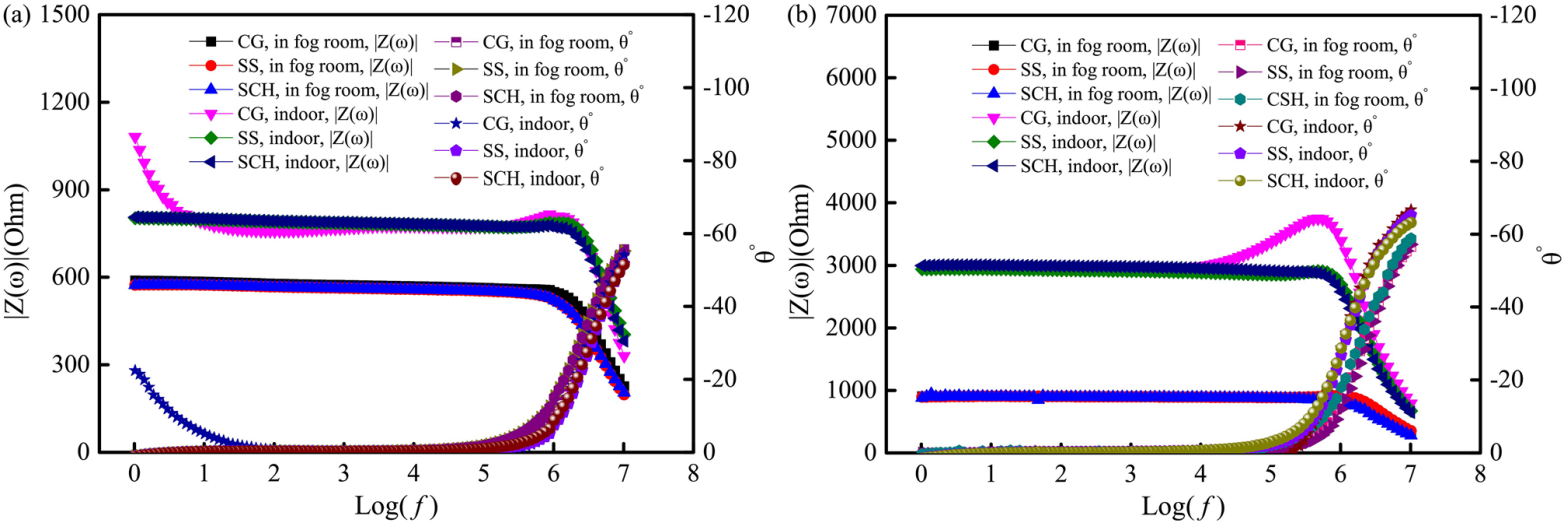
Bode plots of the four-point measurement with different conductive media between the current electrodes and the sample. (**a**) 3d curing ages; (**b**) 28d curing ages.

The Bode plots are shown in Fig. 8. It can be seen from Fig. 8 that the modulus values of ACIS of samples measured by different conductive media and cured in different curing conditions, are almost the same within the range of θ = 0° (low frequency region), and the average modulus values in the range of θ = 0° are shown in Table 3. It can be seen from Table 3 that the CoV of modulus values of ACIS measured by the three conductive media are less than 3.5%, which means that the conductive media have little effect on the low frequency region (θ = 0°) of ACIS measured by the four-point measurement.

**Table 3:** Modulus values of ACIS measured by the four-point measurement under different curing conditions (*L*/*a* = 2).

Curing ages	Conductive media	Fog curing	60% RH curing	Indoor curing	Outdoor curing	Water curing	SCH curing
3 d	CG	1152.6	1743.0	1584.5	1504.4	1125.0	961.6
	SS	1130.1	1678.5	1574.4	1482.1	1104.1	986.4
	SCH	1129.0	1710.8	1581.7	1463.1	1063.7	1046.0
	Average value	1137.2	1710.8	1580.2	1483.2	1097.6	998.0
	CoV (%)	1.0	1.5	0.3	1.1	2.3	3.5
28 d	CG	1806.3	11,063.8	5989.6	3313.6	1343.6	1338.4
	SS	1801.3	10,907.0	5819.5	3273.2	1329.0	1313.1
	SCH	1782.9	11,485.1	5934.3	3248.2	1303.8	1296.7
	Average value	1796.8	11,152.0	5914.5	3278.3	1325.5	1316.1
	CoV (%)	0.6	2.2	1.2	0.8	1.2	1.3

CoV means variation coefficient of test results of three samples measured from the four-point measurement with three conductive media.

As discussed above, depending on the conducive medium, high frequency regions of ACIS measured by four-point measurement may be very different. However, very similar modulus values in the range of θ = 0° (the low frequency region) means that the results measured by the four-point measurement can be used for comparison to those from the two-point measurement, as long as the contact impedance in the range of θ = 0° (the low frequency region) in the four-point measurement is proven to be negligible. Evaluation of the contact impedance in the four-point measurement will be given in “Evaluation of the contact impedance in the four-point measurement” section.

#### Evaluation of the contact impedance in the four-point measurement

Although McCarter et al.^7^ investigated the effect of the contact impedance on two-point measured ACIS in comparison to the four-point measured results, they thought the contact impedance in the four-point measurement was negligible. This point needs to be further investigated. According to the discussion in “Conductive theory of two-point and four-point measurement of ACIS” section, polarization resistance can be negligible for a four-point measurement; however, voltage electrodes need to be precast and thus the extended impedance arises due to current spreading within the specimen in the vicinity of the contact as the electrode size is smaller than the sample section area, these effects were strictly geometric and ohmic and were in no way associated with electrochemical reactions or polarization effect^10,48,49^, additionally, interface impedance truly exists. Therefore, it is necessary to evaluate the effect of the contact impedance on the tested ACIS from the four-point measurement.

The contact impedance in the four-point measurement can be calculated as follows:

Assuming *L*/*a* = *p*, the tested total impedance *Z*_t4_ (*p* = 10) and *Z*_t4_ (*p* = 2) can be expressed as Eqs. (7) and (8):

7 $$Z_{t4} (p = 10) = Z_{im} (p = 10) + Z_{4ic}$$

8 $$Z_{t4} (p = 2) = Z_{im} (p = 2) + Z_{4ic}$$

Equation (9) can be obtained according to Eq. (8):

9 $$5Z_{t4} (p = 10) = 5Z_{im} (p = 10) + 5Z_{4ic}$$

Subtracting Eq. (8) from Eq. (9), Eq. (10) can be obtained:

10 $$5Z_{t4} (p = 10) - Z_{t4} (p = 2) = 5Z_{im} (p = 10) - Z_{im} (p = 2){ + }4Z_{4ic}$$

In theory, Eq. (11) is valid:

11 $$5Z_{im} (p = 10) = Z_{im} (p = 2)$$

Taking Eq. (11) into Eq. (10), Eq. (12) appears:

12 $$5Z_{t4} (p = 10) - Z_{t4} (p = 2) = 4Z_{4ic}$$

Equation (13) can be obtained from Eq. (12):

13 $$Z_{4ic} = \frac{{5Z_{t4} (p = 10) - Z_{t4} (p = 2)}}{4}$$

where the meanings of all of the symbols outside the parentheses are the same as those of the symbols in Fig. 6. The *p* = 2 and *p* = 10 in the parentheses indicate the samples of *L*/*a* = 2 and *L*/*a* = 10, respectively.

**Figure 9:**
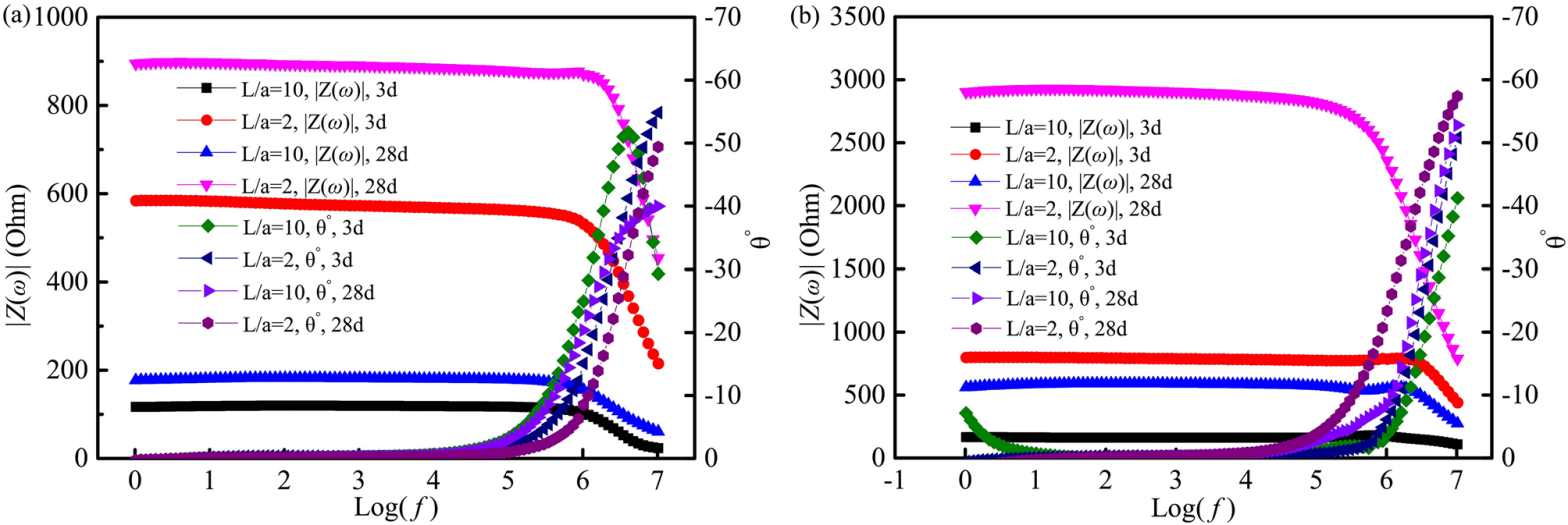
Bode plots of ACIS with *L*/*a* = 2 and 10 measured by four-point measurement. (**a**) In the fog room curing condition; (**b**) Indoor curing condition.

In the case of the four-point measurement, Bode plots of samples exposed to fog curing and indoor environments are given in Fig. 9. Impedance modulus within a frequency range of θ = 0° were calculated based on the data in Fig. 9, and it can be found that the CoV of the modulus values within a range of θ = 0° were less than 1.0%. At the same time, contact impedance in the four-point measurement were calculated based on Eq. (13), the results of which are listed in Table 4. It can be seen from Table 4 that the contact impedance changes from 3.4 to 5.8 Ω for samples cured for 3 days and 28 days in a fog room and in an indoor environment. This means that in this situation, the contact impedance in the four-point measurement is very small, even neglectable, and can be used as a reference for correction of the contact impedance.

**Table 4:** Contact impedance values measured by four-point measurement.

Curing method	Curing age	*L*/*a* = 10			*L*/*a* = 2			*Z*_ic4_ (ohm)
		Z_M10_	*RD*_SCH_ (%)	CoV (%)	Z_M2_	*RD*_SCH_ (%)	CoV (%)	
Fog curing	3d	117.2	4.5	2.8	564.5	0.9	2.2	5.4
	28d	181.0	1.9	1.6	891.5	0.4	1.7	3.4
Indoor Curing	3d	162.7	3.5	2.4	790.9	0.7	1.6	5.7
	28d	598.1	1.0	1.5	2967.2	0.2	0.1	5.8
Oven-drying	48 h	2628.0	32.1	4.4	10,585.6	6.4	2.9	638.6

CoV means variation coefficient of test results of three samples. *RD*_SCH_ means relative deviation of contact impedance using SCH as a conductive media for pure sample impedance. Z_M10_ and Z_M2_ mean the total four-point measurement impedance when *L*/*a* = 10 and 2, respectively.

**Figure 10:**
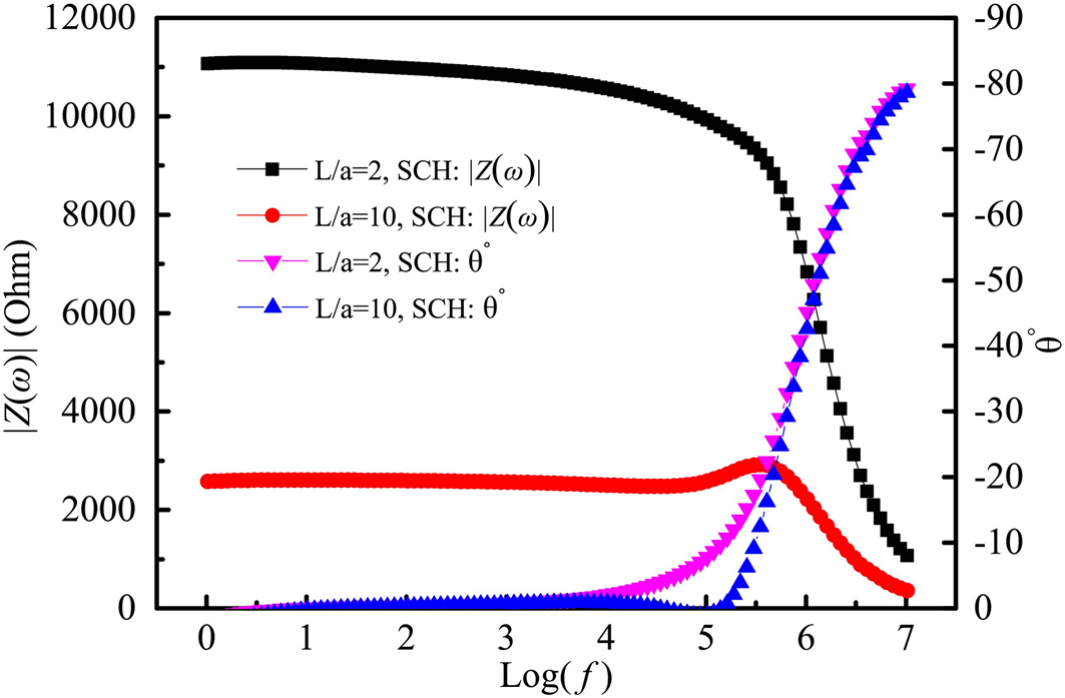
Bode plots of oven-dried samples evaluated by four-point measurement.

The samples cured in an indoor environment for 28 days were dried at 60 °C for 48 h. The Bode plots are shown in Fig. 10. The contact impedance in the four-point measurement was calculated based on Eq. (13), as shown in Table 4. It can be seen from Table 4 that the contact impedance reaches 638.6 Ω, which may be due to an increase in the extended impedance and the interface impedance^10^ as the humidity decreased. The relative deviation caused by the contact impedance reached 32.1% for *L*/*a* = 10, which cannot be used to test the impedance of cement-based materials. While the relative deviation is only 6.4% for *L*/*a* = 2, it can still be used to measure the ACIS of cement-based materials. Therefore, it is reasonable to use *L*/*a* = 2 in this study.

### Evaluation of the contact impedance in the two-point measurement

#### Selection of conductive media in the two-point measurement

**Figure 11:**
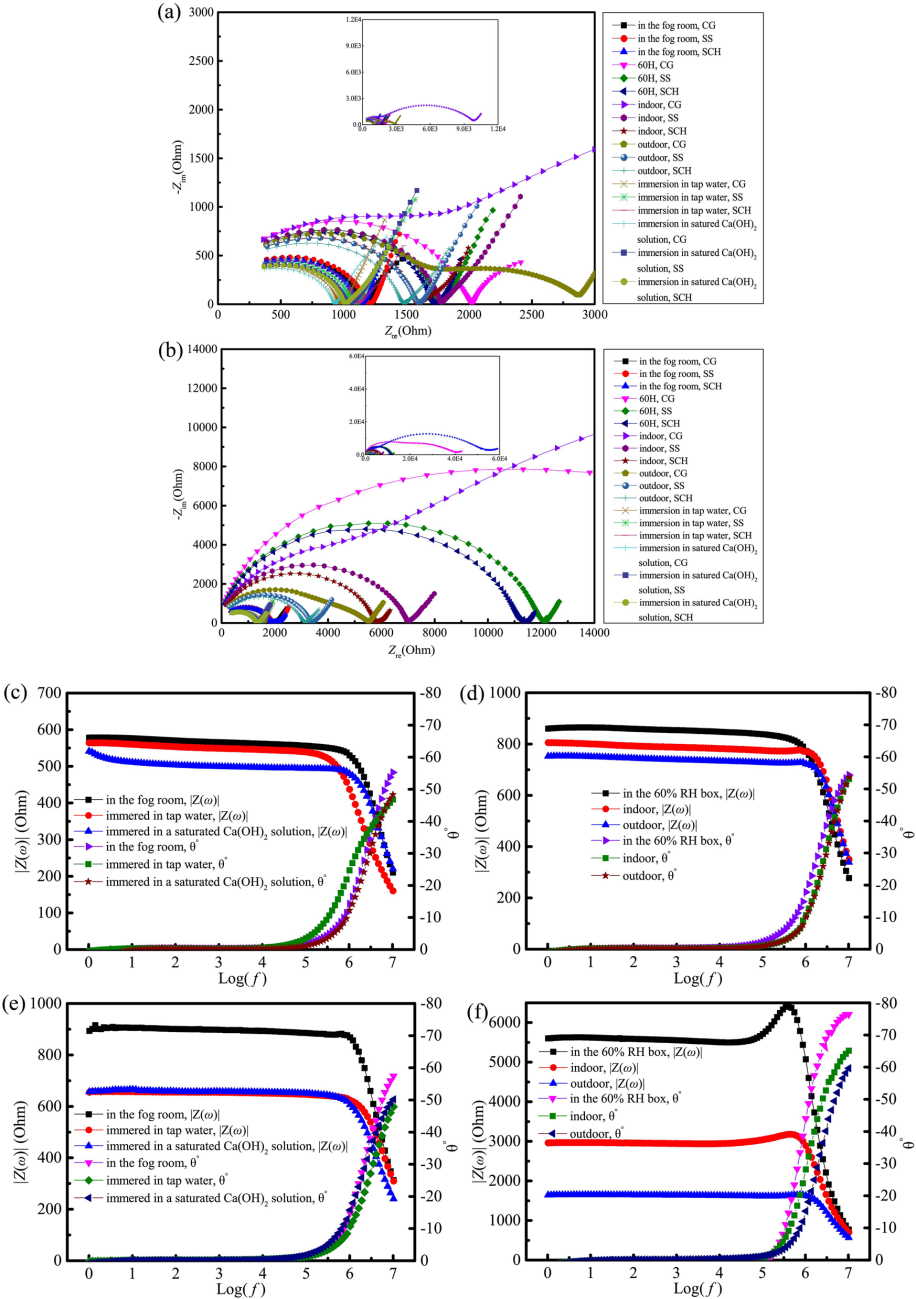
Nyquist plots and Bode plots of samples under different curing conditions. (**a**) Nyquist plots-3d; (**b**) Nyquist plots-28d; (**c**) Bode plots-3d; (**d**) Bode plots-3d; (**e**) Bode plots-28d; (**f**) Bode plots-28d.

Nyquist plots of samples under different curing conditions evaluated by the two-point measurement with different conductive media between the electrodes and the sample are shown in Fig. 11. It can be seen from Fig. 11a,b that even under the same curing condition, the ACISs are significantly different with different conductive media, the diameters of the impedance spectrum arcs are: CG > SS > SCH. According to the generalized effective medium (GEM) theory^50,51^, generally electrical conduction is more likely to take place through the more conductible phases for a composite material with different electrical conductivity for each component^52^. For a cement paste specimen, the conductivity of solid phases (cement clinkers, hydration products) and vapor phases are lower several orders than the liquid phase^53,54^, therefore, the impedance of the specimen is mainly determined by the pore structure and pore solution. When using CG as conductive medium, the charges in the CG are transferred and exchanged with the ions in the pore solution of the specimen under the external voltage^30,31^, However, when using SS or SCH as conductive medium, ions in SS or SCH and the pore solution of the specimen end are more easily transferred, which makes SS or SCH of the more effective conduction rate and ability than that of CG, thus reducing the contact impedance. In the case of drying, the content of the solution at the end of the specimen is less, and this reduction effect is more obvious.

Four-point measurements with *L*/*a* = 2 were conducted for samples cured under different conditions and SCH was filled between the current electrodes and the sample as conductive medium. The Bold plots are given in Fig. 11c,f. This reveals that the modulus values of ACIS measured under different curing conditions have a phase angle of almost 0° in the frequency range of 1 Hz–10^5^ Hz, and the CoV of modulus values is less than 1.0%. That is, |Z(ω)| is approximately equal to the real part value of the sample impedance in this frequency range. Some researchers have pointed out that the impedance at the cut-off frequency in the Nyquist plot measured by the two-point measurement represents the bulk impedance of the cement-based materials^55–58^. In this paper, the impedance modulus values at the corresponding cut-off frequency^1^ in the Nyquist plots were also taken as the bulk impedance of the cement-based materials.

The relative deviation and absolute deviation values of the sample impedance measured by the two- and four-point measurements with different conductive media are shown in Table 5. It can be seen from Table 5 that the *RD*_CG_ of the samples cured for 3 days and 28 days under 60% humidity and indoor curing conditions are 18.5%, 266.5%, 11.9% and 834.3%, respectively. Under outdoor curing condition, they reached 20.9% and 68.9%, respectively, indicating that CG is not suitable for indoor, outdoor, or 60% humidity curing conditions. The *RD*_SS_ of samples cured for 3 days and 28 days under indoor curing condition are 12.6% and 18.5%, respectively. This indicates that SS is no longer suitable for indoor curing condition. Under other curing conditions, the *RD*_SS_ measured by the two-point measurement is in the range of 3.7–8.7%, while under all six curing conditions, the *RD*_SCH_ of contact impedance measured by the two-point measurement is in the range of − 0.8–4.6%, which is less than the *RD*_SS_.

**Table 5:** Relative deviation of sample impedance measured by the two- and four-point measurements under different curing conditions.

Conductive media	CG											
Curing method	Fog curing		60% curing		Indoor curing		Outdoor curing		Water curing		SCH curing	
Curing time	3d	28d	3d	28d	3d	28d	3d	28d	3d	28d	3d	28d
2-point impedance	1173.6	1893.7	2026.6	40,873	1768.6	55,261.0	1792.7	5535.7	1015.7	1433.4	932.0	1355.7
*RD*_CG_ (%)	3.2	5.4	18.5	266.5	11.9	834.3	20.9	68.9	− 7.5	8.1	− 6.6	3.0
*AD*_CG_ (Ω)	36.4	96.9	315.8	29,721.0	188.4	49,346.5	309.5	2257.4	− 121.9	107.9	− 66.0	39.6

Conductive media	SS											
Curing method	Fog curing		60% curing		Indoor curing		Outdoor curing		Water curing		SCH curing	
Curing time	3d	28d	3d	28d	3d	28d	3d	28d	3d	28d	3d	28d
2-point impedance	1211.5	1934.8	1774.4	12,122.4	1779.6	7007.6	1600.6	3399.8	1115.3	1377.1	1062.8	1346.8
*RD*_SS_ (%)	6.5	7.7	3.7	8.7	12.6	18.5	7.9	3.7	1.6	3.9	6.5	2.3
*AD*_SS_ (Ω)	74.3	138.0	63.6	970.4	199.4	1093.1	117.4	121.5	17.7	51.6	114.8	30.7

Conductive media	SCH											
Curing method	Fog curing		60% curing		Indoor curing		Outdoor curing		Water curing		SCH curing	
Curing time	3d	28d	3d	28d	3d	28d	3d	28d	3d	28d	3d	28d
2-point impedance	1133	1879.5	1730.2	11,339.2	1591.7	5821.7	1483.6	3251.3	1082.7	1351.3	1042.9	1313.6
*RD*_SCH_ (%)	− 0.4	4.6	1.1	1.7	0.7	− 1.6	0.0	− 0.8	− 1.4	1.9	4.5	− 0.2
*AD*_SCH_ (Ω)	− 4.2	82.7	19.4	187.2	11.5	− 92.8	0.4	− 27.0	− 14.9	25.8	44.9	− 2.5
4-point impedance	1137.2	1796.8	1710.8	11,152.0	1580.2	5914.5	1483.2	3278.3	1097.6	1325.5	998.0	1316.1

*RD*_CG_, *RD*_SS_ and *RD*_SCH_ mean the relative deviation of contact impedance evaluated by the two-point measurement with different conductive media to the four-point measurement impedance (true sample impedance). *AD*_CG_, *AD*_SS_ and *AD*_SCH_ mean the absolute deviation of contact impedance measured by the two-point measurement with different conductive media versus the four-point measurement impedance (true sample impedance).

It can be seen from Table 5 that under different curing conditions, when the same conductive medium is used, as the curing humidity of the specimen increases, the contact resistance gradually decreases, this indicates that the measured conductivity strongly depends on the curing humidity and conditioned before testing, which coincides with the findings in Reference^59^, and means there are at least two potential influencing factors. On the one hand, the porosity in the cement decreases as the cement hydration rate increases with curing humidity^60,61^, causing the area of the pore in contact with the conductive medium to reduce, which will increase the contact impedance. On the other hand, as the degree of saturation increases, the electron and charge transfer speeds between the end of the test specimens and the conductive medium increase^30,31^, which effectively reduces the contact impedance, it can be seen from the experimental results that the degree of saturation or curing humidity of the specimens plays a major role in the contact impedance, ionic (or electrolytic) conduction is the main phenomenon of electricity transport^62^. Besides, for the specimens cured at the same humidity and using the same medium as the conductive medium, the contact impedance increase with the curing ages, this is due to the porosity and the pore solution conductivity of the cement paste both decrease with the increase of curing age as the cement hydration^63^, which cause greater contact impedance.

#### Effect of oven-drying on the contact impedance in the two-point measurement

**Figure 12:**
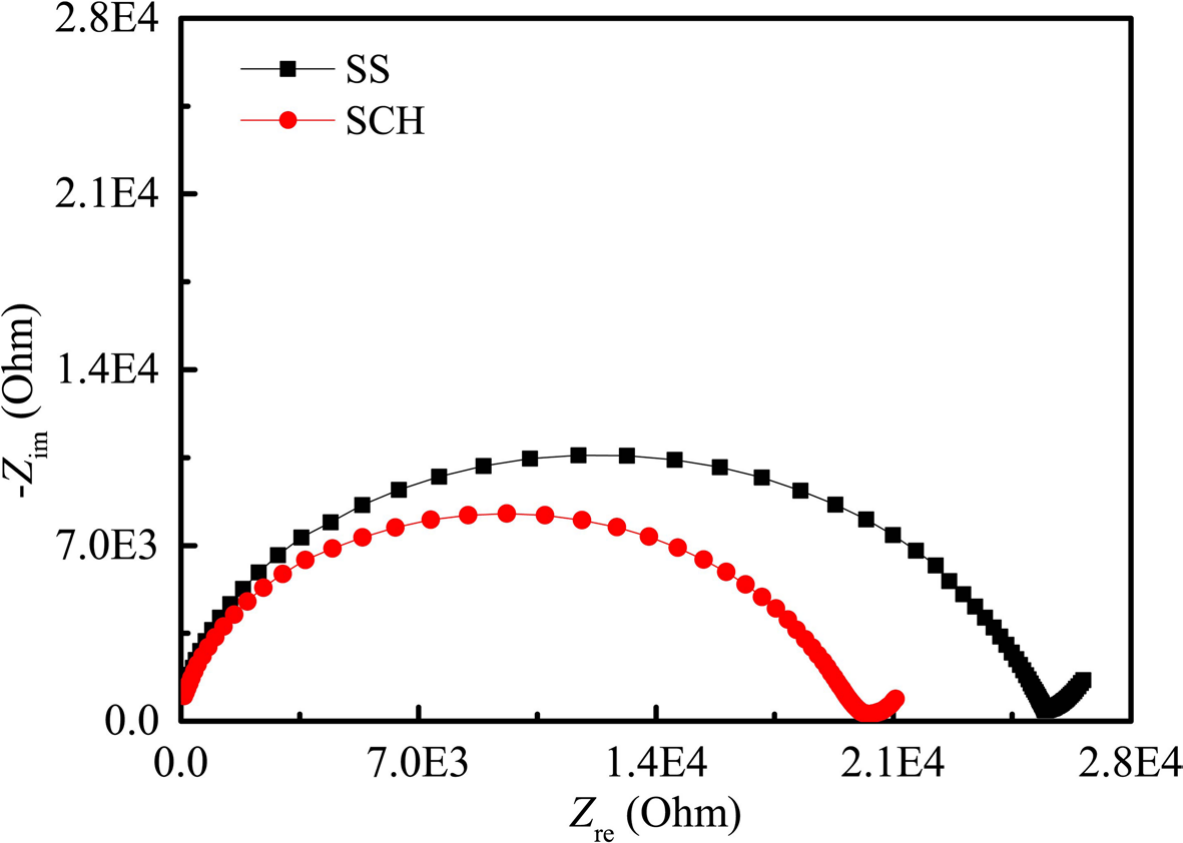
Nyquist plots of oven-dried samples evaluated by two-point measurement.

Two-point measurements were conducted for samples oven-dried for 48 h after curing in an indoor environment for 28 days, using SS and SCH as conductive media between the electrodes and sample, the Nyquist plots are shown in Fig. 12.

The contact impedance in the two-point measurement can be calculated as follows:

*Z*_im_ can be obtained from Eq. (3), as shown in Eq. (14):

14 $$Z_{im} { = }Z_{t4} { - }Z_{4ic}$$

Substituting Eq. (14) into Eq. (1), Eq. (15) can be obtained:

15 $$Z_{m} { = }\frac{L}{a}\left( {Z_{t4} { - }Z_{4ic} } \right)$$

*Z*_2c_ can be obtained from Eq. (2), as shown in Eq. (16):

16 $$Z_{2c} = Z_{t2} - Z_{m}$$

Substituting Eq. (15) into Eq. (16), Eq. (17) can be obtained:

17 $$Z_{2c} = Z_{t2} - \frac{L}{a}\left( {Z_{t4} { - }Z_{4ic} } \right)$$

where the meaning of all symbols are the same as in Fig. 6.

The contact impedance measured by the two-point measurement can be calculated according to Eq. (17), as shown in Table 6. It can be seen from Table 6 that the contact impedance reached 5586.0 Ω and 396.0 Ω in the two-point measurement with SS and SCH as the conductive media, respectively. The *RD*_SS_ reached 28.1%, while the *RD*_SCH_ was only 2.0%; these results mean that under oven-drying conditions, SS is no longer suitable for the two-point measurement, but SCH can still be used as the conductive medium.

**Table 6:** Relative deviation of sample impedance evaluated by two-point measurement with SS and SCH.

Conductive media	Z_t2_ (ohm)	CoV (%)	Pure impedance measured by the four-point measurement (ohm)	*RD* (%)	*AD* (ohm)
SS	25,478.3	10.2	19,894.0	28.1	5586.0
SCH	20,288.3	11.4		2.0	396.0

*RD* means the relative deviation of contact impedance evaluated by the two-point measurement to pure sample impedance. *AD* means absolute deviation of contact impedance evaluated by the two-point measurement to pure sample impedance. CoV means the variation coefficient of the test results of three samples.

From the above discussion, it can be concluded that the contact impedance caused by different conductive media between the electrodes and the sample is mainly affected by the humidity of the sample and the curing conditions. The applicable humidity situations of CG, SS and SCH for the two-point measurement are recommended in Table 7.

**Table 7:** Applicable humidity range of different conductive media for the two-point measurement.

Conductive medium	Relative humidity (%)
CG	> 95
SS	> 60
SCH	–

### Discussion on how to decrease the effect of the contact impedance in the two-point measurement

#### Preparing a reasonable impedance value of the sample based on selection for proper size

As discussed above, it is clearly knowing that the contact impedance cannot be completely avoided in both the four-point and two-point measurements. Even in the recommended curing conditions shown in Table 7, although both of the *RD*_SCH_ and *RD*_SS_ are acceptable for cement-based materials, the *AD*_SCH_ and *AD*_SS_ change within the ranges of 27.2–396.0 Ω and 41.9–970.4 Ω, respectively. This means that when the curing conditions and the conductive media are constant, the reduction in the size of the sample will cause *RD*_SCH_ and *RD*_SS_ to be out of the acceptable range.

Therefore, attention should be paid to assessing the ratio of the contact impedance to the impedance of the sample when measuring ACIS. A larger sample impedance is beneficial to reduce the effect of the relative deviation caused by the contact impedance. It should be pointed out that increasing the length/area (*L*/*A*) to increase the impedance of the sample may cause the tested capacitance to exceed the measurement range of the AC impedance instrument. For example, the measuring ranges of capacitance of HP4194A Impedance/Gain-Phase analyzer and Solartron 1260 impedance analyzer are in the ranges of 10^–14^ to 0.1 F^64^ and 10^–12^ to 0.01 F^65^, respectively. Therefore, in order to minimize the effect of the contact impedance on sample impedance, a reasonable *L*/*A* value should be chosen, which needs further study.

#### Avoiding a large size difference between the electrodes and the sample

**Figure 13:**
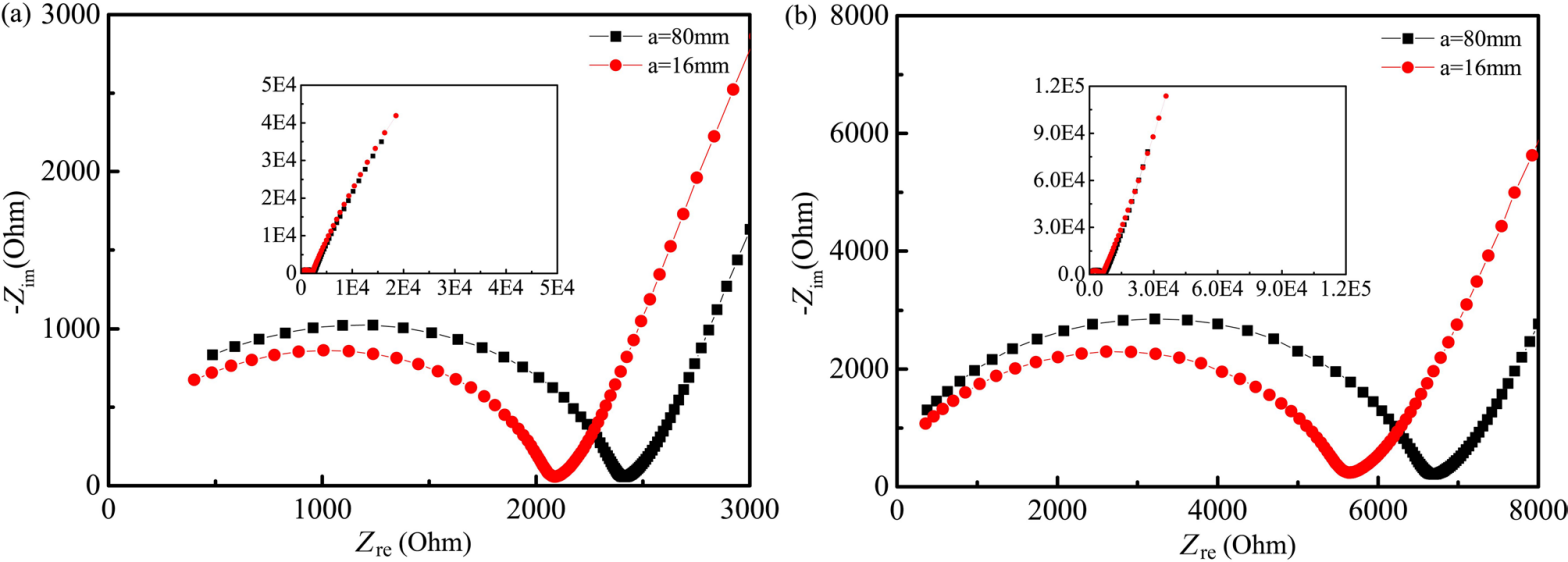
Nyquist plots of oven-dried samples with precast electrodes evaluated by the two-point measurement. (**a**) In the fog room curing condition. (**b**) Indoor curing condition.

The difference in size between the electrodes and sample will result in extended impedance^10^, and extended impedance is especially significant when the electrode is in poor contact with the sample^66^. Two-point measurement with precast titanium bars as both voltage and current electrodes were conducted for samples cured in a fog room and an indoor curing environment for 28 days. The Nyquist plots are shown in Fig. 13. Based on Eq. (13), the contact impedance can be calculated for the two-point measurement with titanium bars as both voltage and current electrodes, as shown in Table 8. It can be seen from Table 8 that the contact impedance of the samples cured in a fog room and in an indoor environment reached 2009.5 and 5390.7 Ω, respectively, resulting in a maximum relative deviation of 2473.2%. Therefore, attention should also be paid to the size difference between the electrodes and sample to avoid a significant effect of the contact impedance.

**Table 8:** Contact impedance evaluated by the two-point measurement with precast titanium bar electrodes.

Curing method	*L*/*a* = 10			*L*/*a* = 2			*Z*’_ic4_ (ohm)
	Z_M10_ (ohm)	*RD* (%)	CoV (%)	Z_M2_ (ohm)	*RD* (%)	CoV (%)	
Fog curing	2090.7	2473.2	14.6	2415.7	494.6	5.0	2009.5
Indoor curing	5651.4	2068.0	7.3	6694.1	413.6	7.3	5390.7

Z_M10_ and Z_M2_ mean the two-point measurement impedance of samples with *L*/*a* = 10 and 2, respectively, with precast titanium bars as both current and voltage electrodes. *Z*’_ic4_ means the contact impedance between the voltage electrodes and the oven-dried sample in the four-point measurement; *RD* means the relative deviation of the contact impedance measured by the two-point measurement to pure sample impedance. CoV means the variation coefficient of the test results of the three samples.

In addition to the above mentioned cases, the relative deviation caused by the contact impedance may not be precisely known, and it is highly recommended in this situation that the four-point measurement with two different *L*/*a* values be conducted to evaluate the effect of the contact impedance on the impedance of cement-based materials using the procedure that has been described in “Theoretical derivation of the relationship among the four-point and two-point measured impedance and the contact impedance” section.

## Conclusions

A method for calculating the contact impedance was proposed in this paper. The contact impedance in two-point and four-point measurements were calculated, and based on the calculated results, the following conclusions can be obtained:

1. The conductive media have a completely different effect on ACIS in the high-frequency region, however, have little effect on the impedance of ACIS in low frequency region (θ = 0°) measured by the four-point measurement.
2. When using the saturated calcium hydroxide solution (SCH) as conductive medium. The contact impedance within a low frequency range of θ = 0° varies from 3.4 to 5.8 Ω in the four-point measurement, which is negligible for the impedance range of cement-based materials. This means that the four-point measurement using SCH as conductive media can be used as a reference for correction of the contact impedance in the two-point measurement.
3. When the samples were dried at 60 °C for 48 h, the contact impedance reaches 638.6 Ω, and the relative deviation caused by the contact impedance reached 32.1% and 6.4% in the four-point measurement for cement paste with *L*/*a* = 10 and *L*/*a* = 2, respectively. This means that a larger sample impedance is beneficial to reduce the effect of the relative deviation caused by the contact impedance in theory. This is also applicable to two-point measurement, but the limitation of the capacitance range of the instrument should be considered.
4. In the case of two-point measurement, under the seven curing conditions, the *RD*_CG_ caused by the contact impedance is in the range of − 7.5–834.3%, *RD*_SS_ is in the range of 2.3–28.1%, and *RD*_SCH_ is in the range of − 0.4–4.6%. In some curing conditions, *RD*_CG_ and *RD*_SS_ are beyond the acceptable range. We found that CG is only suitable for fog room curing and water-curing conditions (RH > 95%), while SS is not suitable for indoor curing and oven-drying conditions (RH < 60%). SCH is suitable for all curing conditions (RH = 0–100%).
5. To reduce the contact impedance caused by the extended impedance, the difference in cross-sectional dimensions between the electrode and the sample should be minimized. In a case of contact impedance not being precisely known, it is highly recommended that a four-point measurement with two different *L*/*a* values should be conducted to evaluate the effect of the contact impedance following the procedure described in “Theoretical derivation of the relationship among the four-point and two-point measured impedance and the contact impedance”.
